# Trenddroge Lachgas (Distickstoffmonoxid, N_2_O) und die Abhängigkeitskriterien nach ICD-10

**DOI:** 10.1007/s00115-024-01736-z

**Published:** 2024-09-23

**Authors:** Dominik Diesing, Peter Neu

**Affiliations:** 1 Klinik für Psychiatrie und Psychotherapie, Charité – Universitätsmedizin Berlin, corporate member of Freie Universität Berlin and Humboldt-Universität zu Berlin, Campus Benjamin Franklin, Hindenburgdamm 30, 12203 Berlin, Deutschland; 2 Klinik für Psychiatrie und Psychotherapie, Jüdisches Krankenhaus Berlin, Berlin, Deutschland

**Keywords:** Distickstoffmonoxid, Nebenwirkungen, Abhängigkeit von psychoaktiven Substanzen, Drogenmissbrauch, Psychologie, Neurologische Manifestationen, Drogenkonsum, Jugendliches Gesundheitsverhalten, Nitrous oxide, adverse effects, Substance-related disorders, psychology, Substance abuse, psychology, Neurological manifestations, drug effects, Adolescent behavior, drug effects

## Abstract

**Fragestellung:**

Die steigende Beliebtheit von Lachgas (Distickstoffmonoxid, N_2_O) als Freizeitdroge wirft Fragen nach seinem Abhängigkeitspotenzial auf. Dieses narrative Review untersucht das Abhängigkeitsrisiko von N_2_O anhand der ICD-10-Kriterien für Abhängigkeitserkrankungen und bewertet die aktuelle Literatur.

**Material und Methoden:**

Eine umfassende Literatursuche bis April 2024 wurde durchgeführt, um Publikationen zu identifizieren, die den Konsum von N_2_O im Kontext von Abhängigkeitskriterien behandeln. Die Ergebnisse wurden anhand der ICD-10-Kriterien analysiert.

**Ergebnisse:**

Studien zeigen gemischte Ergebnisse hinsichtlich des Cravings und Kontrollverlusts bei N_2_O‑Konsument:innen. Es gibt Hinweise auf Vernachlässigung anderer Interessen und mögliche Toleranzentwicklung, während die Daten zu Entzugssymptomen begrenzt sind. Ein anhaltender Konsum trotz schädlicher Folgen wurde beschrieben, jedoch fehlen objektive diagnostische Methoden zur Bestimmung der Konsumintensität.

**Diskussion:**

Die Datenlage zum Abhängigkeitspotenzial von N_2_O ist uneinheitlich. Die Diskussion um seine Klassifizierung als abhängigkeitserzeugende Substanz bleibt kontrovers. Klinische Hinweise deuten jedoch auf ein mögliches Abhängigkeitsrisiko hin, insbesondere bei exzessivem Konsum.

**Fazit:**

Lachgas wird gegenwärtig vor allem als Missbrauchssubstanz betrachtet und hat das Potenzial, bei exzessivem Konsum eine psychische Abhängigkeit zu fördern, die sich insbesondere durch Kontrollverlust und Vernachlässigung äußert. Die Kriterien für eine körperliche Abhängigkeit, wie das Auftreten eines Entzugssyndroms und die Entwicklung von Toleranz, sind bisher jedoch noch nicht ausreichend überzeugend dokumentiert worden. Weitere Forschung ist erforderlich, um das Abhängigkeitspotenzial von N_2_O besser zu verstehen und angemessene präventive und therapeutische Maßnahmen zu entwickeln.

## Abstract (en)

**Objective:**

The increasing popularity of nitrous oxide (N_2_O) as a recreational drug raises questions about its potential for dependency. This narrative review examines the dependency risk of N_2_O using the ICD-10 criteria for substance use disorders and evaluates the current literature.

**Material and methods:**

A comprehensive literature search until April 2024 was conducted to identify publications addressing N_2_O consumption in the context of dependency criteria. The results were analyzed based on the ICD-10 criteria.

**Results:**

Studies showed mixed results regarding craving and loss of control among N_2_O users. There is evidence of neglect of other interests and potential tolerance development, while data on withdrawal symptoms are limited. Persistent consumption despite harmful consequences has been described but objective diagnostic methods to determine consumption intensity are lacking.

**Discussion:**

The data on the dependency potential of N_2_O are inconsistent. The discussion on its classification as an addictive substance remains controversial; however, the clinical indications suggest a possible risk of dependency, especially with excessive consumption.

**Conclusion:**

Nitrous oxide is currently primarily regarded as a substance of abuse with the potential to foster psychological dependence, manifesting particularly through loss of control and neglect; however, the criteria for physical dependence, such as the occurrence of withdrawal syndromes and the development of tolerance, have not yet been convincingly documented. Further research is needed to better understand the dependency potential of N_2_O and develop appropriate preventive and therapeutic measures.

## Hintergrund

Die steigende Beliebtheit von Distickstoffmonoxid (N_2_O; auch als Lachgas bekannt) als Freizeitdroge hat in der medizinischen Forschung und Praxis zu verstärktem Interesse geführt.

N_2_O wurde ursprünglich als Anästhetikum in der Medizin und Zahnmedizin sowie als Treibmittel in der Lebensmittelindustrie eingesetzt. Heutzutage hat sich der Gebrauch durch Inhalation von N_2_O (auch als „hit“ bezeichnet) aufgrund seiner euphorisierenden Effekte auch im Freizeitbereich etabliert [23]. Wie ein aktueller Bericht der WHO konstatiert, ist die Halbwertszeit von N_2_O im menschlichen Körper mit typischerweise etwa 5 bis 15 min sehr kurz, der Konsum wird häufig bagatellisiert [16]. N_2_O birgt trotz der oft wahrgenommenen Harmlosigkeit ernsthafte Risiken für die physische und psychische Gesundheit.

Obwohl in der Literatur bisher keine akuten schwerwiegenden oder lebensbedrohlichen Komplikationen im Rahmen eines Freizeitmonokonsums beschrieben werden, kann exzessiver Konsum langfristig zu neurologischen Schädigungen führen, wie beispielsweise Myeloneuropathie, Parästhesien und weitere diffuse fokalneurologische Defizite, hauptsächlich aufgrund eines Vitamin-B12-Mangels. Während des akuten Rausches können psychische Störungen wie Angstzustände, Halluzinationen und Dissoziationen auftreten. Ebenfalls scheint das Risiko für Atemdepressionen durch eine Mischintoxikation mit Alkohol anzusteigen, die Datenlage bezüglich vital bedrohlicher Komplikationen durch einen Mischkonsum mit anderen Substanzen ist unzureichend [7, 27, 29].

Dawudi et al. [5] untersuchten in der Region um Paris zwischen 2018 und 2021 in einer multizentrischen Kohortenstudie die Inzidenz von schweren N_2_O‑induzierten neurologischen Störungen. Die Ergebnisse zeigten, dass 25 % der 181 stationär neurologisch und internistisch behandelten Konsument:innen eine Myelopathie, 37 % eine periphere Neuropathie und 38 % ein gemischtes Symptombild aufwiesen, welche die Autor:innen am ehesten auf einen konsumbedingten Vitamin-B12-Mangel zurückführten. Besonders besorgniserregend in dieser Studie ist ebenfalls der steile Anstieg der Inzidenz von N_2_O‑induzierten neurologischen Störungen ab 2020, der Mitte 2021 mit 6,15 pro 100.000 Personen seinen Höhepunkt erreichte. Dies ist vergleichbar mit der Inzidenz der Multiplen Sklerose (MS), welche in Europa je nach Region zwischen 2 und 10 pro 100.000 Personen pro Jahr beträgt [12]. Diese wachsende Anzahl an Folgeerscheinungen wirft die Frage auf, wie hoch das Abhängigkeitspotenzial dieser Substanz zu bewerten ist, da offensichtlich ein nicht unerheblicher Teil der Konsument:innen trotz bereits eingetretener gesundheitlicher Probleme den Konsum fortsetzt.

Seit Mitte der 2010er-Jahre ist eine starke Zunahme der Anzahl wissenschaftlicher Artikel zu beobachten, die sich mit den Risiken und dem Missbrauchspotenzial von Lachgas auseinandersetzen [2, 7]. Die Zunahme ist auf die Erforschung neuer Indikationen bei psychischen Störungen sowie auf die wachsende Popularität von N_2_O als Rauschmittel zurückzuführen. Insbesondere unter Jugendlichen und jungen Erwachsenen ist der Konsum stark gestiegen [24].

Das European Monitoring Centre for Drugs and Drug Addiction (EMCDDA) berichtete im Jahr 2023, dass N_2_O aktuell als eine der am weitesten verbreiteten psychoaktiven Substanzen in Europa gilt. Die Prävalenz des missbräuchlichen Konsums von Lachgas (N_2_O) unter jungen Erwachsenen in Deutschland hat in den letzten Jahren ebenfalls zugenommen. Laut dem European Drug Report der EMCDDA lag die Prävalenz des Konsums unter 15- bis 34-Jährigen im Jahr 2021 bei etwa 2,7 %. Im Jahr 2020 waren es etwa 1,8 %, und 2019 lag die Prävalenz noch bei etwa 1,1 % [19].

In den sozialen Medien und auf den Onlineportalen überregionaler deutschsprachiger Nachrichtenmagazine werden vermehrt Beiträge veröffentlicht, die den Konsum von N_2_O durch Jugendliche thematisieren. Dabei werden oft leere N_2_O‑Flaschen an öffentlichen Orten wie Straßenrändern, Spielplätzen oder Parkplätzen gezeigt [21, 22].

Viele klinisch tätige Psychiater:innen betrachten den zunehmenden Konsum unter Jugendlichen als große Gefahr für die Entwicklung einer Abhängigkeitserkrankung [1, 7, 25]. Einige Autor:innen insbesondere neurobiologischer Untersuchungen hingegen gehen jedoch aufgrund fehlender Merkmale, die typischerweise mit Abhängigkeitserkrankungen einhergehen (Entzugssymptome, Toleranzentwicklung, Aktivierung des mesolimbischen Pathways), eher von einem geringen bis fehlenden Abhängigkeitspotenzial aus [4, 6, 9]. Personen mit exzessivem Konsumverhalten werden in der Regel eher in Einzelfallstudien oder Fallserien als in systematischen Erhebungen beschrieben.

Die Studienlage an randomisierten placebokontrollierten Untersuchungen zu diesem Thema ist zwar begrenzt, jedoch wird in einem kürzlich erschienenen Review von Back et al. [1] das Abhängigkeitspotenzial von N_2_O anhand der DSM-5-Kriterien für eine Substanzkonsumstörung bewertet. Die Autor:innen sind sich sicher, dass bei einem Großteil der Menschen mit exzessivem Konsummuster hauptsächlich vier der DSM-5-Kriterien erfüllt sind (Kriterium 1, 3, 6 und 8). Dies entspricht einer mittelschweren Substanzkonsumstörung. Vergleiche mit der in Deutschland gebräuchlichen Internationalen Klassifikation der Krankheiten fehlen jedoch bislang.

In dieser narrativen Übersichtsarbeit wird die aktuelle wissenschaftliche Literatur zum Konsum von N_2_O und dessen Abhängigkeitsrisiko untersucht und anhand der in Deutschland verbreiteten ICD-10-Kriterien i. S. einer Abhängigkeitserkrankung bewertet.

## Methodik

Eine umfassende Literatursuche wurde in der elektronischen Datenbank PubMed durchgeführt, um Forschungsarbeiten zu identifizieren, die den Konsum von N_2_O im Kontext der ICD-Kriterien für Abhängigkeitserkrankungen thematisieren. Die Suche konzentrierte sich auf Publikationen bis April 2024. Die Suchstrategie umfasste eine Kombination der Begriffe „nitrous oxide + dependence“, „nitrous oxide + substance use disorder“, „nitrous oxide + addiction“, „nitrous oxide + abuse“ und „nitrous oxide + case report“.

Die Suche führte zur Identifizierung von insgesamt 73 relevanten Publikationen. Von diesen wurden 13 Reviews (hiervon 3 systematische Reviews), 14 klinische Fallstudien und 16 Fallberichte ausgewählt, um einen umfassenden Überblick über die aktuellen Erkenntnisse zu geben. Metaanalysen zu diesem Thema sind uns derzeit nicht bekannt.

Die Studienauswahl erfolgte nach strengen Einschlusskriterien: Es wurden nur Artikel in Peer-reviewed-Journals berücksichtigt, die in englischer oder deutscher Sprache veröffentlicht wurden und die sich spezifisch mit dem schädlichen Gebrauch/Missbrauch sowie dem Abhängigkeitspotenzial von N_2_O befassten. Veröffentlichungen, die sich ausschließlich auf die medizinische Anwendung von Lachgas ohne Bezug zum Missbrauch oder zur Abhängigkeit konzentrierten, wurden ausgeschlossen. Insgesamt umfasste die Analyse 43 Arbeiten, die eine fundierte Grundlage für die weiteren Ausführungen dieses Reviews darstellen.

Anhand der ICD-10-Kriterien (Tab. 1) wird im Folgenden die aktuelle Datenlage bewertet.

**Tab. 1:** Kriterien für ein Abhängigkeitssyndrom nach ICD-10, Hinweise für das Vorliegen des jeweiligen Kriteriums sowie eine Einordnung in Bezug auf die Fragestellung

	Kriterium nach ICD-10^a^	Evidenz	Bewertung
1	Ein starker Wunsch oder eine Art Zwang, die Substanz zu konsumieren (Craving)	Während Tierstudien nur einen geringen Verstärkungswert andeuten, berichten Humanstudien und Fallberichte über intensives Verlangen bei einigen Konsument:innen. Besonders intensive Nutzer:innen berichten von einem starken Verlangen, das durch die Verabreichung von Naltrexon gemildert werden konnte [14], was darauf hinweist, dass Craving bei einem Teil der N_2_O‑Konsument:innen vorhanden ist	Nicht klar belegt, widersprüchliche Ergebnisse
2	Verminderte Kontrollfähigkeit bezüglich des Beginns, der Beendigung und der Menge des Konsums	Studien wie das systematische Review von Fidalgo et al. [6] zeigen, dass über 98 % der Konsument:innen N_2_O häufiger und in größeren Mengen konsumieren als beabsichtigt. Dies deutet auf eine signifikante Schwierigkeit hin, den Konsum zu kontrollieren, was auf eine verminderte Kontrollfähigkeit hinweist	Scheint erfüllt
3	Körperliches Entzugssyndrom bei Beendigung oder Reduktion des Konsums, Konsum derselben Substanz (oder einer nah verwandten Substanz), um Entzugssymptome zu lindern oder zu vermeiden	Bisherige Untersuchungen an Tiermodellen und Schilderungen aus Case-Reports konnten keine vegetativen Entzugssymptome nachweisen [7]. Intensive Nutzer:innen berichten zwar von einem „unangenehmen Gefühl“ nach abklingender Wirkung, jedoch fehlen systematische Studien, die ein spezifisches Entzugssyndrom beim Menschen bestätigen	Eher nicht erfüllt, systematische Untersuchungen fehlen
4	Nachweis einer Toleranz. Um die ursprünglich durch niedrigere Dosen erreichten Wirkungen zu erzielen, sind zunehmend höhere Dosen erforderlich	Während eine ältere Studie zeigt, dass der analgetische Effekt mit zunehmender Konsumhäufigkeit nachlässt [26], gibt es keine klaren Hinweise auf eine Toleranzentwicklung gegenüber den euphorisierenden Effekten. In den meisten Fallberichten wurde kein ausgeprägter Toleranzeffekt festgestellt, und die pro „hit“ inhalierte Dosis muss nicht notwendigerweise gesteigert werden, um den gleichen Effekt zu erzielen [10, 28]	Nicht klar belegt
5	Vernachlässigung anderer Vergnügen oder Interessen zugunsten des Substanzgebrauchs; erhöhter Zeitaufwand, um die Substanz zu beschaffen, zu konsumieren oder sich von ihren Wirkungen zu erholen	Die prospektive Beobachtungskohortenstudie von Nugteren-Van Lonkhuyzen et al. [15] berichtet, dass 9 von 10 intensiven N_2_O‑Konsument:innen andere Interessen vernachlässigten und signifikant viel Zeit für den Erwerb und Konsum von N_2_O sowie die Erholung davon aufwandten. Diese Beobachtungen deuten darauf hin, dass dieses Kriterium bei vielen intensiven N_2_O‑Nutzer:innen erfüllt ist	Scheint erfüllt
6	Anhaltender Substanzgebrauch trotz des Nachweises eindeutig schädlicher Folgen, wie Leberschädigung durch exzessives Trinken, depressive Verstimmungen infolge starken Substanzkonsums usw., wobei die/der Betroffene sich dieser Art und dem Umfang der Schädigung bewusst sind, oder es wird davon ausgegangen, dass er/sie sich in Anbetracht seines/ihres Wissens darüber bewusst sein müsste	Laut dem Global Drug Survey von 2014 setzten etwa 4 % der Konsument:innen trotz anhaltender Parästhesien den Konsum fort, was auf eine Ignorierung der schädlichen Effekte hinweist. Zudem berichten Fallstudien über Konsument:innen, die trotz neurologischer und psychiatrischer Folgeschäden weiterhin N_2_O konsumieren [10, 29]. Diese Fälle deuten darauf hin, dass einige Konsument:innen ihren Gebrauch trotz bekannter schädlicher Konsequenzen fortsetzen	Scheint teilweise erfüllt, systematische Untersuchungen fehlen

^a^Für die Diagnose eines Abhängigkeitssyndroms nach ICD-10 müssen mindestens drei Kriterien während des letzten Jahres gleichzeitig vorhanden gewesen sein

## Ergebnisse

### Craving

Studien zur Selbstverabreichung von N_2_O in Tierexperimenten deuten darauf hin, dass N_2_O einen geringen Verstärkungswert besitzt und kein starkes Verlangen hervorruft [18]. Allerdings zeigen Humanstudien zur Motivation, N_2_O im Vergleich zu einem Placebo zu konsumieren, gemischte Ergebnisse. In einer Studie bevorzugten 80 % der Nichtdrogenkonsument:innen, die an der Studie teilgenommen haben, N_2_O dosisabhängig gegenüber Sauerstoff. Eine ähnliche Studie ergab lediglich bei 22 % der Fälle eine Bevorzugung von N_2_O gegenüber dem Placebo [1, 4]. In Einzelfallberichten von intensiven Konsument:innen wird jedoch ein starkes Verlangen berichtet, welches durch die Verabreichung von Naltrexon erfolgreich gemildert wurde [14].

Die Hinweise für einen dosisabhängigen verstärkenden Effekt von N_2_O sind unklar und es gibt nur begrenzte Belege, hauptsächlich aus nichtrandomisierten Fallstudien.

### Kontrollverlust

Fidalgo et al. [6] beschreiben in ihrem systematischen Review das Suchtverhalten bei N_2_O‑Konsument:innen. Die in dieser Arbeit eingeschlossenen Studien zeigen, dass über 98 % der Konsument:innen N_2_O in größeren Mengen und über längere Zeiträume als beabsichtigt verwenden. Ein solches Verhaltensmuster kann ein Indiz für einen Kontrollverlust sein. Die betroffene Person wird zunehmend unfähig, ihren Konsum einzuschränken, trotz des Wissens um mögliche schädliche Auswirkungen, wie weiter unten beschrieben.

Es ist jedoch zu beachten, dass die Fallzahl bei allen diesen Studien gering ist und größere (mindestens *N* > 100) randomisierte Untersuchungen bislang fehlen.

### Vernachlässigung anderer Interessen

Die Datenlage ist ebenfalls uneinheitlich. Es gibt jedoch Fallberichte von Konsument:innen, die durch intensiven Zeitaufwand rund um die Substanz starke negative soziale Folgen erfahren haben. In einer prospektiven Beobachtungskohortenstudie von Nugteren-Van Lonkhuyzen et al. [15] berichten 9 von 10 niederländischen Patient:innen, die exzessiv N_2_O konsumierten, von einer Vernachlässigung anderer Interessen zugunsten des Konsums. Trotz Bemühungen, den Konsum zu kontrollieren, erlebten einige dieser Konsument:innen wiederholt Rückfälle. Aktuelle Untersuchungen liefern gemischte Ergebnisse. Zusätzlich zum Kontrollverlust, der oben erwähnt wurde, betonen Back et al. in ihrem Review auch die erhebliche Zeit, die von einigen „heavy users“ für den Erwerb und Gebrauch von N_2_O aufgewendet wird. In einigen Fallserien berichten über 90 % der Fälle von signifikanten Zeitaufwendungen in diesem Kontext [1].

Die nur begrenzt vorliegenden Daten deuten darauf hin, dass zumindest ein Teil der N_2_O‑Nutzer:innen eine erhebliche Zeit damit verbringt, N_2_O zu erwerben, zu konsumieren und sich von den Auswirkungen zu erholen. Dies könnte auf das Vorliegen dieses Kriteriums einer Substanzgebrauchsstörung hinweisen.

### Toleranz

Es gibt bislang keine direkten Untersuchungen zur Toleranz im Rahmen der erwünschten euphorisierenden Effekte. Jedoch lässt der analgetische Effekt mit zunehmender Konsumhäufigkeit nach, wie eine bereits fast 50 Jahre alte Studie zeigt [26]. Es ist jedoch wichtig zu beachten, dass dies nicht unmittelbar auf eine Toleranz gegenüber der erwünschten Wirkung übertragbar ist. In den meisten Fallberichten wurde kein ausgeprägter Toleranzeffekt im Selbstbericht festgestellt [28]. Es gibt Hinweise darauf, dass die pro „hit“ inhalierte Dosis nicht unbedingt gesteigert werden muss, um den gleichen Effekt zu erzielen [10].

Eine mögliche alternative Erklärung für das Nachlassen der analgetischen Wirkung könnten neurochemische Veränderungen im Gehirn sein, die durch häufigen Gebrauch verursacht werden und die Reaktion auf Schmerz anders beeinflussen als die Reaktion auf euphorische Stimuli. Systematische Untersuchungen hierzu fehlen bislang. Zukünftige Studien sollten sich darauf konzentrieren, wie und warum bestimmte Wirkungen der Substanz eine Toleranzentwicklung zeigen, während andere dies weniger tun. N_2_O wirkt schmerzstillend, indem es die Aktivität von NMDA-Rezeptoren im zentralen Nervensystem moduliert [11]. Bei wiederholtem Gebrauch könnte es zu einer Anpassung dieser Rezeptoren kommen, was zu einer verminderten Schmerzempfindlichkeit führt. Die euphorisierende Wirkung von N_2_O wird vor allem durch die Freisetzung von Dopamin im Belohnungssystem des Gehirns vermittelt [3]. Dieses System könnte weniger anfällig für eine schnelle Toleranzentwicklung der Substanz sein, da es auf unterschiedliche Weise reguliert wird.

### Entzugssymptome

Bisherige Untersuchungen an Tiermodellen konnten keine vegetativen Entzugssymptome feststellen. Jedoch berichten intensive Nutzer:innen von einem „unangenehmen Gefühl“ mit abklingender akuter Wirkung, wie es vermutlich bei den meisten euphorisierend wirkenden Substanzen üblich ist [7]. Es gibt nach unserer Kenntnis keine Untersuchung zu Entzugssymptomen beim Menschen.

### Anhaltender Konsum trotz schädlicher Folgen

Ein Problem bei der Bewertung dieses Symptoms besteht darin, dass ein Teil der Nutzer:innen sich möglicherweise der potenziell schädlichen Auswirkungen eines intensiven Konsums nicht bewusst ist. Während der Konsum bei den meisten Freizeitkonsument:innen sporadisch und zeitlich begrenzt auftritt, gibt es eine Minderheit, bei der ein exzessives Konsummuster vorliegt. Hierbei besteht eine Korrelation zwischen der Anzahl der „hits“ und dem Ausmaß an fokalneurologischen Defiziten [29]. Laut dem Global Drug Survey von 2014 setzten etwa 4 % der Konsument:innen trotz anhaltender Parästhesien den Konsum fort. Beschreibungen von Konsument:innen, die trotz direkter negativer erheblicher gesundheitlicher Folgen ein anhaltendes Konsummuster aufweisen, finden sich eher in Fallberichten. In diesen Fällen lag meistens eine polyvalente Substanzkonsumstörung vor [10].

### Objektivierbare Diagnostik bezüglich Konsumfrequenz und -intensität

Die objektive Erfassung der Konsumhäufigkeit und -frequenz stellt eine besondere Herausforderung dar, da die Substanz nach der Inhalation innerhalb von Minuten nahezu vollständig eliminiert wird. Derzeit werden Patient:innen, bei denen der Verdacht auf aktiven N_2_O‑Missbrauch besteht, anhand von indirekten Indikatoren wie Vitamin B12, Homocystein und Methylmalonsäure identifiziert. Diese Biomarker stehen in Zusammenhang mit den Auswirkungen von N_2_O auf den Stoffwechsel [13], sind jedoch unspezifisch und liefern keinen Beweis für einen chronischen Konsum.

## Diskussion

Die Debatte über das Abhängigkeitspotenzial von Distickstoffmonoxid (N_2_O), auch bekannt als Lachgas, ist durch eine bislang unzureichende und uneinheitliche Datenlage geprägt. Die Annahme, dass Substanzen mit Abhängigkeitspotenzial eine gemeinsame neurobiologische Route über das mesokortikolimbische Dopaminsystem aktivieren, welche typischerweise bei Abhängigkeitserkrankungen zu finden sind, findet bei N_2_O bislang keine eindeutige Bestätigung. Hier ist jedoch zu erwähnen, dass es sich größtenteils um ältere Untersuchungen handelt, die teils über 50 Jahre zurückliegen. Während Tierstudien eher kein Verstärkungspotenzial durch N_2_O aufzeigen und Humanstudien gemischte Ergebnisse liefern, lassen neuere Untersuchungen und Fallserien auf mögliche dopaminerge Mechanismen durch N_2_O schließen [1, 2, 8]. Brunt et al. [4] schlussfolgern, dass N_2_O aufgrund seiner kurzen Halbwertszeit vermutlich nicht die langanhaltenden Veränderungen im mesokortikolimbischen System hervorruft, wie sie von anderen Substanzen bekannt sind. Parallelen lassen sich hier jedoch zu einer anderen Substanz ziehen, welche trotz ebenfalls kurzer Halbwertszeit von bis zu 60 min ein sehr hohes Abhängigkeitspotenzial aufweist, nämlich GBL/GHB, sodass dies unserer Meinung nach eher kein Argument für eine geringe Abhängigkeitswahrscheinlichkeit liefert [30]. Vielmehr scheint es die rasche Anflutung im Gehirn zu sein, die die Höhe des Abhängigkeitspotenzials bestimmt. Diese dürfte aufgrund des Konsumweges (inhalativ) eher für das Vorliegen eines hohen Abhängigkeitspotenzials sprechen.

Die von Back et al. postulierte Evidenz für Craving, negative soziale Konsequenzen, einen erheblichen Zeitaufwand für den Gebrauch und die Beschaffung sowie einen Konsum in größeren Mengen als beabsichtigt ist größtenteils qualitativer oder kasuistischer Natur. Die Verbindung von N_2_O‑Konsum mit risikoreichem Verhalten junger Menschen und die möglichen körperlichen Schäden durch Mangel an Vitamin B12 unterstreichen in jedem Fall die Existenz eines schädlichen Gebrauchs mit potenziell gravierenden gesundheitlichen Schäden bei exzessivem Konsum.

Lachgas wird gegenwärtig vor allem als Missbrauchssubstanz betrachtet und hat das Potenzial, bei exzessivem Konsum eine psychische Abhängigkeit zu fördern, die sich insbesondere durch Kontrollverlust und Vernachlässigung äußert. Die Kriterien für eine körperliche Abhängigkeit, wie das Auftreten eines Entzugssyndroms und die Entwicklung von Toleranz, sind bisher jedoch noch nicht ausreichend überzeugend dokumentiert worden. Dem trägt das DSM‑5 bereits Rechnung und klassifiziert den Substanzkonsum unter „inhalant use disorder“ [17]. In Großbritannien und den Niederlanden existiert bereits ein Verbot des Besitzes von N_2_O ohne entsprechende Lizenz [20].

Als Limitation dieser Arbeit ist zu erwähnen, dass diese Befunde größtenteils nicht auf prospektiven oder sogar randomisiert kontrollierten Beobachtungsstudien basieren, sondern hauptsächlich aus Fallserien hervorgehen.

Dennoch ist die Auseinandersetzung mit den Grundlagen der Risikobewertung für eine Abhängigkeitserkrankung und die kritische Neubewertung von Substanzen eine lohnende Unternehmung, auch vor dem Hintergrund der Neudefinition der Abhängigkeitskriterien im ICD-11. Die zukünftige Forschung sollte daher darauf abzielen, die neuropharmakologischen Phänomene der Abhängigkeit im Kontext von N_2_O und anderen Substanzen tiefergehend zu verstehen und die Datenlage sowohl in qualitativer als auch quantitativer Hinsicht zu erweitern. Diese Bemühungen sind dringend notwendig, um zu einer fundierten Beurteilung des Abhängigkeitspotenzials von N_2_O zu gelangen und effektive präventive sowie therapeutische Maßnahmen zu entwickeln.

## Fazit für die Praxis

- Insbesondere bei jüngeren Patient:innen mit einer Myelopathie oder peripheren Neuropathie und Vitamin-B12-Mangel sollte auch nach möglichem N_2_O‑Substanzkonsum gefragt werden.
- Angesichts der aktuell limitierten Evidenz und der epidemiologischen Daten zu einer steigenden Zahl von Personen mit Folgeschäden aufgrund von N_2_O‑Konsum erscheint es angebracht, den Konsum von N_2_O zur Erzielung eines euphorisierenden Effektes derzeit zumindest als schädlichen Gebrauch zu behandeln.
- Der belastete Begriff der Abhängigkeit sollte vorerst vermieden werden bis überzeugendere Belege vorliegen.
- Eine Verharmlosung des Konsums sollte in Anbetracht der steigenden Anzahl von N_2_O‑Konsument:innen mit neurologischen oder psychiatrischen Folgeschäden unbedingt vermieden werden.
